# Effectiveness of Early Intervention Programs for Young Children with Global Developmental Delay: A Systematic Review -

**DOI:** 10.31661/gmj.vi.3906

**Published:** 2025-07-29

**Authors:** Sarah Mohammed Almuhanna, Omar Ahmed Alshehri, Bandar Mohammed B. alsaadoon, Mohammad Hassan Mohammed Alawad, Abdullah Mohammad Alnasyan, Salman Abdullah Abuabat, Myle Akshay Kiran

**Affiliations:** ^1^ Pediatric Department, King Saud Bin Abdulaziz University for Health Sciences University, Riyadh, Saudi Arabia; ^2^ Department of Scientific Clinical Research and Pharmacology, Hospital and Health care Administration, Acharya Nagarjuna University, India; ^3^ General and Alternative Medicine, National Institute of Medical Science, Pratista, Andhra Pradesh, India; ^4^ Ceo of Medsravts in Education and Clinical research, Andhra Pradesh, India

**Keywords:** Global Developmental Delay, Early Intervention, Neurodevelopmental Disorders, Parent-mediated Therapy, Child Development, Rehabilitation, Systematic Review

## Abstract

**Background:**

Global Developmental Delay (GDD) affects cognitive, language, motor, and adaptive functions in children under five years and is often a precursor to long-term neurodevelopmental disorders including intellectual disability and autism spectrum disorder. Early intervention can improve developmental outcomes, yet evidence regarding its effectiveness, especially in low-resource and diverse clinical settings, remains fragmented. To synthesize available evidence on the effectiveness of early intervention programs for children aged 0–6 years with GDD, and to identify intervention types, outcomes, and gaps in current research.

**Materials and Methods:**

A systematic review following PRISMA 2020 guidelines was conducted. PubMed, Web of Science, Scopus, and ClinicalTrials.gov were searched up to October 14, 2025, using predefined search terms. Eligible studies included children aged 0–6 years diagnosed with GDD or nonspecific developmental delay and involved an early intervention program assessing developmental outcomes. Data extraction and quality appraisal were performed using Joanna Briggs Institute (JBI) tools. Narrative synthesis was used due to heterogeneity across studies.

**Results:**

Six studies involving a total of 689 participants were included. Interventions varied widely, including multidisciplinary rehabilitation, parent-mediated programs, community-based approaches, and combined medical–therapeutic methods. All studies reported improvements in at least one developmental domain (motor, language, cognitive, or social), with greater gains observed when interventions were initiated early (6 months) and sustained over longer durations. Parent-mediated and community-based models were feasible and effective in low-resource settings. However, no randomized controlled trials were identified, and most studies showed moderate to high risk of bias. Certainty of evidence was rated low to very low using GRADE.

**Conclusion:**

Early intervention programs demonstrate consistent benefits for children with GDD across settings, particularly when initiated early and involving caregivers. However, the current evidence base is limited by methodological weaknesses and lack of standardized outcome measures. High-quality randomized trials and long-term follow-up studies are urgently needed to inform best practices and policy implementation.

## Introduction

Early childhood developmental delays (ECDDs) include a spectrum of conditions where a child’s cognitive, motor, social, or language development lags behind typical milestones, influenced by genetic, environmental, and socioeconomic factors [1][2]. Global developmental delay (GDD) is a condition marked by significant delays in two or more developmental areas—such as language, motor, cognitive, or social skills, before age five [3]. Causes are diverse and include genetic factors, metabolic disorders, and environmental influences [4]. Early evaluation with genetic testing, hearing and vision screening, and metabolic assessment is essential to identify treatable conditions and guide intervention [3]. Many children with GDD later develop intellectual disability and frequently show comorbid autism spectrum disorder, especially when language delays are severe [5]. Early, multidisciplinary intervention can improve long-term functional outcomes [6]. These delays, potentially stemming from conditions like autism spectrum disorder (ASD) or environmental issues such as prenatal toxin exposure and poverty, significantly impact long-term cognitive, emotional, and social outcomes [2][7][8].

Early detection through tools like the Ages and Stages Questionnaires (ASQ) and Modified Checklist for Autism in Toddlers (M-CHAT) is critical, as timely interventions can mitigate developmental challenges and promote equitable opportunities [9][10][11]. The heterogeneity in diagnostic approaches, varying by geography and healthcare systems is evident in literature. But there are widely accepted precise methods like the Bayley Scales of Infant and Toddler Development (Bayley-III) and Autism Diagnostic Observation Schedule (ADOS-2), which offer high sensitivity for early identification [12][13][14]. The brain’s rapid growth in the first five years is the reason for the urgency of early intervention to optimize developmental trajectories and reduce long-term issues like academic underachievement and social difficulties [15][16]. Based on early diagnosis, proper interventions can be applied. Interventions for ECDDs, including Applied Behavior Analysis (ABA), speech-language therapy, and Early Childhood Education (ECE) programs, are most effective when intensive and initiated before age three, significantly improving cognition, language, and social skills [17][18][19]. ABA, widely used for ASD, enhances communication and academic functioning, while the Early Start Denver Model (ESDM) integrates play-based strategies for social and cognitive gains [20][21][22]. Speech-language therapy addresses communication delays, reducing frustration and fostering social relationships, and occupational therapy supports motor skill development for daily activities [23][24]. ECE programs, such as Head Start, provide structured environments that boost cognitive and language development, particularly for disadvantaged children [25][26]. Multidisciplinary and parent-mediated interventions further enhance outcomes by tailoring treatments to individual needs, aligning with guidelines from the American Academy of Child and Adolescent Psychiatry for personalized, early approaches [27][28][29]. In case of GDD, there is limited synthesis of evidence specifically examining the effectiveness of early intervention programs for young children with GDD, especially within diverse clinical, community, and low-resource settings where implementation barriers are greatest. Therefore, this study is essential to systematically evaluate existing intervention approaches, identify evidence gaps, and guide policy and practice toward equitable, early developmental support for at-risk children.

## Materials and Methods

### Study Design

This systematic review was conducted in accordance with the Preferred Reporting Items for Systematic Reviews and Meta-Analyses (PRISMA 2020) guidelines [30]. A narrative synthesis approach was used to summarize and integrate findings on early intervention for young children with GDD.

### Search Strategy

A structured literature search was carried out in PubMed, Web of Science, Scopus, and clinicaltrials.gov to identify peer-reviewed studies evaluating early intervention programs for children diagnosed with GDD or developmental delays. The search strategy combined controlled vocabulary (MeSH) and free text terms related to developmental delay, intervention, and child populations:

("global developmental delay" OR "developmental delay" OR "developmental disabilities"

OR "neurodevelopmental delay")

AND ("intervention" OR "therapy" OR "early intervention" OR "rehabilitation")

AND ("outcome" OR "effectiveness" OR "developmental progress")

AND ("child" OR "infant" OR "preschool")

Filters: English language; publication date up to October 14, 2025.

Reference lists of included studies were manually screened to identify additional eligible articles. No other databases were searched due to time and resource constraints.

### Eligibility Criteria

Studies were included if they involved children aged 0-6 years diagnosed with global developmental delay or nonspecific developmental delay and evaluated any early intervention program, such as multidisciplinary rehabilitation, family-based programs, community programs, parent-mediated programs, or combined medical and therapeutic approaches. Eligible studies could use any control group, including usual care or waiting lists, or a pre-post design without a control, and had to report at least one developmental domain outcome, including motor, language, cognitive, social, or adaptive functioning. Acceptable study designs included quasi-experimental, cohort, observational, or comparative clinical studies, and only studies published in English were considered. Studies were excluded if they involved children older than six years, lacked an intervention component, were case reports, conference abstracts, letters, or commentaries, or did not report measurable developmental outcomes.

### Study Selection

Search results were imported into a reference manager and screened in two stages. First, titles and abstracts were independently screened by two reviewers using predefined eligibility criteria. Full-text screening was then performed for potentially relevant articles. Disagreements at either stage were resolved through discussion or by consulting a third reviewer.

### Data Extraction

A structured data extraction form was used to capture study characteristics, including author, year, country, setting, and sample size; participant characteristics, such as age and diagnosis; study design and comparator; intervention type and delivery format, for example, parent-mediated, center-based, or community-based; follow-up duration; outcome measures, including DQ scores, Griffiths Scales, CGAS, and parenting stress; and key findings. Data extraction was conducted by one reviewer and subsequently checked by a second reviewer.

### Quality Appraisal

Methodological quality of included studies was assessed using the Joanna Briggs Institute (JBI) critical appraisal tools for quasi-experimental and observational studies [31]. Risk of bias domains included selection bias, comparability, reliability of outcome measurement, and completeness of follow-up. Evidence certainty across studies was later assessed using the GRADE framework [32].

### Data Synthesis

Due to heterogeneity in study design, interventions, and outcome measures, a meta-analysis was not feasible, so a narrative synthesis was used to summarize the evidence. The synthesis was organized by intervention type, including multidisciplinary, parent-mediated, community-based, and medical plus rehabilitation approaches; developmental outcomes, such as motor, cognitive, language, and social/adaptive domains; and follow-up duration. Additionally, consistency of findings, methodological limitations, and the overall strength of evidence were evaluated. PRISMA flow chart was generated by an online tool [33].

## Results

**Table:** Table2. JBI Appraisal of Included Studies

**Study (first author, year / source) **	**Country / setting **	**Design**	**N (analyzed) **	**Age (months) **	**Intervention (description) **	**Comparator / control **	**Outcome measures **	**Duration / follow-up **
Fang Li et al. [34]	China (single centre; hospital + home)	Prospective clinical comparison (non-randomised)	120 (90 experimental, 30 control)	Not explicitly stated (young children)	Individualized inpatient treatment **+ acupuncture** combined with home-based intervention therapy	Home-based intervention only (no inpatient treatment)	Developmental Quotients (DQ) across domains: gross motor, fine motor, adaptability, language, personal-social; clinical effective rate	Not fully specified (pre/post; implied single treatment episode)
Al-Yamani et al. [35]	Arab Gulf region (not further specified)	Observational retrospective with comparative group	NR (sample size not given in excerpt)	3-6 years (preschool children)	Structured therapeutic program (preschool day treatment centre program) delivered ≥1 academic year	Waiting-list / control group	Children’s Global Assessment Scale (CGAS); placement into integrated regular classes	At least one academic year (pre/post)
Dan Liu et al. [36]	China (Hebei children’s hospital)	Retrospective cohort	155 infants (all diagnosed; analyses by age group)	3-18 months (groups: 3-6, 7-12, 13-18 mo)	Comprehensive rehabilitation therapy (based on Gesell assessments)	Pre-post within-subject (no parallel control described)	Gesell Developmental Schedules (GDS) — DQ in adaptive, gross motor, fine motor, language, personal-social domains	≥3 months of therapy (pre and post)
Ping Dong et al. [37]	China — multicentre (several children’s/rehab hospitals)	Multicentre controlled intervention (likely quasi-experimental)	306 total (153 experimental, 153 control)	3-6 months at enrollment	Parent-Implemented Early Intervention Program (PIEIP) delivered to parent-child dyad	Control: usual care / waiting list (not receiving PIEIP)	Griffiths Mental Development Scale-Chinese (GDS-C): locomotor, personal-social, language, general quotient (GQ); parenting stress scales	Assessments at 12 and 24 months of age (midterm & end)
Liu Xiumei et al. [38]	China (treatment = family + hospital)	Observational / treatment vs control	Treatment 45, Control 30	Not explicitly stated — children with GDD (likely toddler/preschool range)	Portage Guide to Early Education (PGEE): family + hospital combined, 1:1	Control: usual care / no PGEE	Gesell Infant Development Scale (GESELL) DQ; Social-Life Abilities Scales (SM)	6 months (pre and 6-month post)
Lakhan et al. [39]	India (NGO / community-based rehabilitation in tribal setting)	Program evaluation; pre-post within-subjects	67 enrolled; 46 completers analyzed	6-36 months	Early intervention delivered by Community-Based Rehabilitation Workers (CBRWs) under professional supervision; low-cost local materials	No parallel control (pre-post)	Early Intervention Tool (EIT) covering motor, communication, cognitive, social domains	Duration not explicitly stated in excerpt; pre-post (follow-up until compliance)

**Table:** Table2. JBI Appraisal of Included Studies

**Study**	**Design**	**JBI Questions (Key) **	**Appraisal Summary **
**Fang Li et al., 2024 ** [34]	Quasi-experimental	1. Were the participants similar? 2. Was there a control group? 3. Were outcomes measured reliably? 4. Was follow-up complete? 5. Were outcomes assessed in a blinded way?	1. Yes - groups comparable at baseline. 2. Yes - experimental vs home-based control. 3. Yes - developmental quotients and clinical effectiveness. 4. Mostly - 7/127 excluded. 5. Not reported - possible bias.
**Al-Yamani et al., 2023 [35] **	Observational comparative	1. Were groups comparable? 2. Were outcomes measured consistently? 3. Were confounders identified? 4. Was follow-up complete?	1. Partially - some baseline differences possible. 2. Yes - CGAS scoring. 3. Limited - some confounders acknowledged. 4. Not fully reported - retrospective design limits follow-up assessment.
**Liu et al., 2025 ** [36]	Retrospective cohort	1. Were groups similar? 2. Were exposures measured reliably? 3. Were outcomes measured validly? 4. Was follow-up adequate?	1. Yes - age-based stratification. 2. Yes - intervention clearly described. 3. Yes - GDS pre/post assessment. 4. Adequate - all received ≥3 months therapy.
**Dong et al., 2023 ** [37]	Multicenter quasi-experimental	1. Were participants comparable? 2. Was intervention clearly described? 3. Were outcomes measured reliably? 4. Was follow-up complete? 5. Was there statistical analysis?	1. Yes - age and baseline DQ similar. 2. Yes - PIEIP intervention described. 3. Yes - DQ and GDS-C scores, parental stress. 4. Yes - mid-term and end-term assessments. 5. Yes - t-tests and significance reported.
**Liu et al., 2018 ** [38]	Observational comparative	1. Were groups similar at baseline? 2. Were outcomes measured validly? 3. Was intervention described? 4. Was follow-up complete?	1. Yes - baseline DQ comparable. 2. Yes - GESELL DQ and social adaptability. 3. Yes - PGEE program described. 4. Yes - 6-month follow-up for all analyzed.
**Lakhan et al., 2013 ** [39]	Observational pre-post	1. Were participants described? 2. Were outcomes measured reliably? 3. Was follow-up adequate? 4. Was statistical analysis appropriate?	1. Yes - age, DD severity reported. 2. Yes - Early Intervention Tool (EIT). 3. Mostly - only 46/67 analyzed. 4. Yes - paired t-test, correlation analysis.

**Figure-1:**
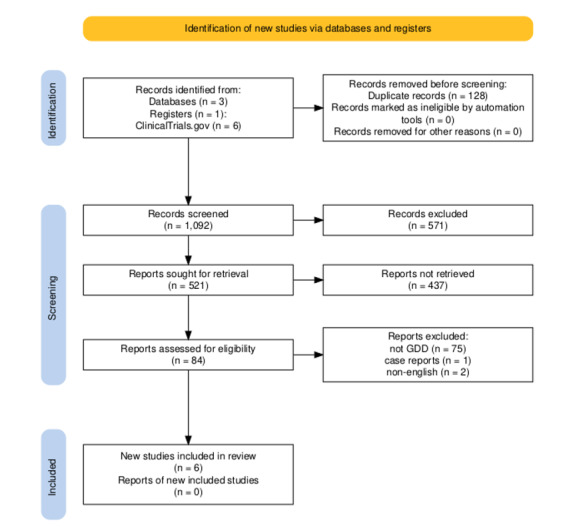
Figure 1. PRISMA flowchart of inclusion process of studies

**Figure-2:**
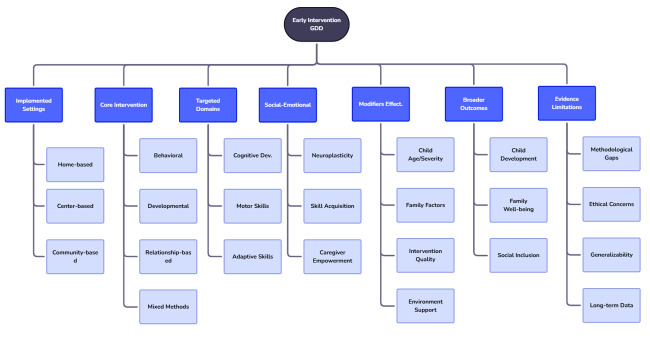
Figure 2. Summary of study findings, generated by visily.ai

A total of 1,092 records were identified through database searching and screened by title and abstract. After removing duplicates and applying the initial screening, 84 full-text articles were assessed for eligibility. Following detailed evaluation against the predefined inclusion and exclusion criteria, six studies were finally included in this systematic review, as shown in Figure-1.

Across the six included studies, sample sizes ranged from small single-center cohorts of 46 to moderately large multicenter samples exceeding 300 participants, reflecting variability in study scale and methodological rigor. Most studies were conducted in clinical or rehabilitation settings in Asia, particularly China and India, with one study from the Arab Gulf region. Participant ages ranged from infancy to early childhood, typically between 3 months and 6 years, aligning with the critical period for neurodevelopmental intervention. Study designs were predominantly observational and quasi-experimental, including retrospective cohorts, pre-post intervention designs, and controlled comparison groups, with no randomized controlled trials identified among the included evidence. Intervention approaches varied widely, encompassing multidisciplinary rehabilitation programs, parent-implemented therapy, structured educational models, community-based interventions, and combined medical-therapeutic methods such as acupuncture. Control conditions, where reported, generally involved routine care, waiting-list groups, or home-based intervention without structured clinical support. Follow-up durations ranged from three months to one academic year, though many studies lacked long-term outcome assessment.

Outcome measures were heterogeneous across studies, but most utilized standardized developmental assessment tools to examine change in developmental functioning. Common measures included the Gesell Developmental Schedules (GDS), Developmental Quotient (DQ) across functional domains, Griffiths Mental Development Scales, social adaptability assessments, and global functioning scales such as the CGAS. Despite heterogeneity in tools and domains assessed, all studies evaluated multidimensional aspects of child development, particularly gross and fine motor skills, language, adaptability, and social interaction. A small number of studies also incorporated caregiver-related outcomes such as parental stress, reflecting broader psychosocial impacts of intervention. However, reporting quality varied, with frequent lack of detail regarding intervention fidelity, treatment intensity, and attrition analysis. Several studies also lacked clear descriptions of baseline comparability or adjustment for confounding variables, limiting interpretability.

Figure-2 shows summarized synthesized evidence. Across the included studies, early intervention consistently demonstrated positive developmental trends in children with GDD, although the strength of this evidence is limited by study design. Multidisciplinary rehabilitation and medical-therapeutic approaches, such as combined acupuncture and home-based therapy, showed improvements in multiple developmental domains (Li et al. 2024). Parent-mediated interventions also demonstrated measurable benefits, particularly when caregiver training was central to the therapeutic model (Dong et al. 2020). Structured early education programs delivered through family-clinic collaboration similarly enhanced developmental quotients across domains (Liu et al. 2018), while community-based programs in low-resource settings successfully improved motor, cognitive, and communication abilities using low-cost materials (Lakhan et al. 2013). Observational cohort data further indicated that earlier initiation of intervention (especially before 6 months of age) may yield greater treatment responsiveness (Liu et al. 2024), and longer intervention exposure (e.g. one academic year) may lead to enhanced functional integration outcomes such as readiness for school (Al-Yamani et al. 2023). Despite consistent directionality favoring early intervention, methodological heterogeneity, lack of randomisation, and incomplete adjustment for confounders limit causal interpretation across studies.

Using the GRADE framework, certainty of evidence for core developmental outcomes remains low. Improvements were reported in motor, language, and adaptive/social functioning across multiple studies (Li et al. 2024; Dong et al. 2020; Liu et al. 2018; Lakhan et al. 2013), but mostly from observational or quasi-experimental designs without blinding, introducing risk of bias. Consistency of effect direction was moderate, but precision was reduced by small sample sizes in some studies (Liu et al. 2018; Lakhan et al. 2013) and indirectness was present due to variation in intervention content and delivery across populations and settings (community vs. hospital-based). Only one study incorporated parent-related outcomes, demonstrating reductions in parental stress linked to intervention participation (Dong et al. 2020), but evidence remains sparse for family or quality-of-life outcomes. Overall, the certainty of evidence is graded as low to very low, indicating that while findings are promising and support implementation of early intervention programs, further high-quality controlled studies are necessary to increase confidence in estimated effects.

### Quality of Included Studies

Across studies, interventions were generally well-described, outcomes reliably measured, and improvements consistently observed, but limitations included small control groups, potential baseline differences, lack of blinding, and partial loss to follow-up. Table 2 summarizes appraisal results in studies.

## Discussion

The findings of our review are consistent with previous literature demonstrating that early intervention yields measurable improvements in motor, language, cognitive, and social domains among children with developmental delays. Orton et al. (2024) [40], in a Cochrane systematic review of early developmental programs for high-risk infants, reported small but significant improvements in motor and cognitive outcomes when interventions were started early and delivered consistently.

However, unlike these prior reviews that often-included heterogeneous populations such as children with cerebral palsy, autism, and general developmental risk, our study focused specifically on children diagnosed with GDD, addressing a notable gap in the literature by examining intervention outcomes in this clinically distinct group.

Similarly, Dong et al. (2023) [41] found that parent-mediated and multidisciplinary interventions significantly improved functional developmental outcomes, particularly in language and social communication, highlighting the importance of caregiver involvement. In agreement with our findings, Smythe et al. (2021) [42] emphasized that community-based and family-centered early intervention models are feasible and effective in low- and middle-income countries, even where access to specialized rehabilitation services is limited.

Consistent with earlier findings, the current body of evidence continues to be limited by a lack of randomized controlled trials, short follow-up durations, and inconsistent use of standardized developmental assessment tools across studies.

In comparison to prior syntheses, such as Kumar et al., which analyzed 14 studies in low- and middle-income countries showing parent-led interventions improved cognitive/language outcomes [43]; Emmers et al., a meta-analysis of 19 studies (n=19,765) in rural China demonstrating parental training reduced delay risk [44]; and Aldharman et al. [45], reviewing 13 studies where early diagnosis/intervention doubled developmental gains across NDD domains (communication had most benefit). Like these studies, we showed greater gains with interventions initiated before 6 months and sustained over time, while indicting persistent methodological limitations, including the absence of randomized controlled trials and low-to-very low GRADE evidence certainty, echoing calls for high-quality, standardized trials and long-term follow-ups.

## Conclusion

Early interventions for children with global developmental delay consistently transformed neurodevelopmental trajectories across diverse low-resource settings, empowering caregivers as pivotal agents of change while illuminating the profound urgency of intervening before six months to harness brain plasticity's critical window. Yet, the pervasive absence of randomized trials and methodological fragilities reveal a fragile evidence foundation that risks perpetuating inequitable care, demanding immediate investment in rigorous, standardized research to translate promising gains into equitable policy and scalable global programs.

## Conflict of Interest

None.
